# Reproduction of Tissue by Skin Grafting

**Published:** 1886-07

**Authors:** 


REPRODUCTION OF TISSUE BY SKIN GRAFTING.—By Wm. H. Atkinson, M. D., D. D. S., New York. This is the substance of an address before the New York Odontological Society.

